# Innate immunity ascertained from blood and tracheal aspirates of preterm newborn provides new clues for assessing bronchopulmonary dysplasia

**DOI:** 10.1371/journal.pone.0221206

**Published:** 2019-09-04

**Authors:** Patrizia Zaramella, Fabio Munari, Matteo Stocchero, Barbara Molon, Daniel Nardo, Elena Priante, Francesca Tosato, Luca Bonadies, Antonella Viola, Eugenio Baraldi

**Affiliations:** 1 Neonatal Intensive Care Unit, Department of Women’s and Children’s Health, Padova University Hospital, Via Giustiniani, Padova, Italy; 2 Department of Biomedical Sciences, University of Padova, Padova, Italy; 3 Pediatric Research Institute (IRP), Città della Speranza Foundation, Padova, Italy; 4 Department of Laboratory Medicine, Padova University Hospital, Padova, Italy; Hospital for Sick Children, CANADA

## Abstract

**Aim:**

The study aimed to establish how granulocytes, monocytes and macrophages contribute to the development of bronchopulmonary dysplasia (BPD).

**Materials and methods:**

Study A: samples of blood and tracheal aspirates (TAs) collected from preterm newborn infants during the first 3 days of life were investigated by flow cytometry, and testing for white blood cells (WBCs), neutrophils and neutrophil extracellular traps (NETs). Maternal blood samples were also collected. Study B: data from previously-tested samples of TAs collected from preterm newborn infants were re-analyzed in the light of the findings in the new cohort.

**Results:**

Study A: 39 preterm newborn infants were studied. A moderate correlation emerged between maternal WBCs and neutrophils and those of their newborn in the first 3 days of life. WBCs and neutrophils correlated in the newborn during the first 8 days of life. Decision rules based on birth weight (BW) and gestational age (GA) can be used to predict bronchopulmonary dysplasia (BPD). Neutrophil levels were lower in the TAs from the newborn with the lowest GAs and BWs. Study B: after removing the effect of GA on BPD development, previously-tested newborn were matched by GA. Monocyte phenotype 1 (Mon1) levels were lower in the blood of newborn with BPD, associated with a higher ratio of Monocyte phenotype 3 (Mon3) to Mon1. Newborn infants from mothers with histological chorioamnionitis (HCA) had lower levels of classically-activated macrophages (M1) and higher levels of alternatively-activated macrophages (M2) in their TAs than newborn infants from healthy mothers.

**Conclusion:**

Immune cell behavior in preterm newborn infants was examined in detail. Surprisingly, neutrophil levels were lower in TAs from the newborn with the lowest GA and BW, and no correlation emerged between the neutrophil and NET levels in TAs and the other variables measured. Interestingly, monocyte phenotype seemed to influence the onset of BPD. The rise in the ratio of Mon 3 to Mon 1 could contribute to endothelial dysfunction in BPD.

## Introduction

Among the pathophysiological factors implicated in bronchopulmonary dysplasia (BPD), the inflammasome and immunity have been attracting the interest of investigators in the field of preterm newborn research. Maternal choriodecidual membranes and lung inflammatory response both seem to play a part as well, judging from findings in tracheal aspirates (TAs) from preterm newborn infants [1].

The human immune system originates from the yolk sac and then develops in a process that continues throughout pregnancy and is completed after birth [2]. Neutrophils are the most abundant type of granulocyte and white cell in mammals. They are the phagocytes found in the bloodstream, driven towards the interstitium by chemotaxis triggered by chemokines and leukotrienes. In addition to recruiting and activating other cells in the immune system, neutrophils have a key role in the front-line defense against invading pathogens, environmental agents, or cancer. Neutrophils have three ways to attack micro-organisms directly: phagocytosis (ingestion), degranulation (release of soluble anti-microbials), and the generation of neutrophil extracellular traps (NETs) [3]. In neonates NETs are released from neutrophils in response to fungal stimuli [4], but many proteins associated with the neutrophil death known as NETosis are downregulated in cord neutrophils [5], an important factor that helps to explain the newborn’s greater exposure to infections. NETosis induces the delivery of fibrous NET structures formed mainly of nuclear DNA with its associated histones, and nuclear, cytoplasmic, and granular proteins. NETs exert pro-inflammatory effects on the epithelial airways, but they are also capable of resolution in crystal-mediated inflammation [6, 7]. In fact, after neutrophils have accomplished their immunological action, their apoptosis is essential to the regression of inflammation or tissue damage, whereas the prolonged survival of neutrophils usually aggravates the injury. Li et al. [8] demonstrated a pathogenic role of NETs in ventilator-induced lung injury, that we assume might be partially similar to the progression of lung injury towards chronic disease in infants born preterm, though no data exist as yet on the role of NETs in BPD.

Intra-amniotic infection and histological chorioamnionitis (HCA) are inflammation-related conditions characterized by a rise in white blood cells (WBCs) and neutrophils in the choriodecidual membranes and blood of the affected newborn [9–11]. Neutrophils contribute to tissue repair by promoting the release of angiogenic VEGF, by cutting off tissue debris, by feeding forward macrophages, or by apoptosis [12]. Cooperation between neutrophils and macrophages [13] seems to create a connection between innate and adaptive immune responses. Several deprivation experiments have demonstrated the fundamental role of both neutrophils and macrophages in acquired defenses against infectious pathogens [14]. The monocyte-macrophage system was previously elucidated in a group of premature babies at our center [15]. We measured monocytes (Mon) in the blood, and classically-activated macrophages (M1 phenotype, or CAM), and alternatively-activated macrophages (M2 phenotype, or AAM) in TAs. Mon/M1 exhibited phenotype CD14++/CD16-, Mon/M2 were intermediate CD14++/CD16+, and Mon/M3 were CD14+/CD16++. An impaired classical activation pathway was found in infants born at the earliest gestational ages (GAs). In addition, the newborn who would subsequently develop BPD revealed lower levels of M1, and higher levels of M2 at birth [15].

The aim of the present study was to investigate the role of the main cells of innate immunity in the development of BPD. The study is divided into two parts. First, a cohort of preterm newborn infants was enrolled to investigate the relationships between their blood and tracheal aspirates and their white blood cells, neutrophils, neutrophil extracellular traps and maternal data (study A). Then data collected in our previous study [15] were re-analyzed to examine the relationships between the onset of BPD and the monocyte-macrophage phenotype, without the confounding effect of GA (study B).

## Methods

### Experimental design

The study was conducted with the approval of the Ethics Committee of Padova University General Hospital, first granted on 12 November 2012, and updated on 22 September 2016 (Prot. n. 2724P). Signed informed consent forms were obtained from all participating parents. The research was conducted according to the principles expressed in the Declaration of Helsinki.

Study A was a prospective observational study designed to examine the relationship between WBC counts in the bloodstream and TAs from newborn, and to correlate their levels with maternal variables (chorioamnionitis, eclampsia, type of delivery) and neonatal variables including GA, birthweight (BW), BPD, early sepsis, and levels of cells (neutrophils and granulocytes), and enzymes in TAs. Newborn infants admitted to the NICU at the Department of Women’s and Children’s Health at the University of Padova from January 2016 to December 2017 were identified from a prospectively-managed database. Infants with major congenital malformations were not considered eligible for enrollment.

Study B investigated the relationships between blood and TA flow cytometric data and outcomes in terms of BPD, HCA and patent ductus arteriosus (PDA), without the confounding effect of GA. Data obtained from previously-tested newborn infants were re-examined considering groups of patients matched by GA. More details about the data collection and analytical methods can be found in Milan et al. [15].

### Study A

#### Sample collection

Blood samples were routinely collected from the umbilical catheter on admission to the NICU or within 3 days to obtain baseline hematological parameters, and then non-invasively as part of routine newborn clinical surveillance and/or hematological data monitoring.

To obtain the TAs the airways were suctioned with 0.5 mL of 0.9% saline injected through a tracheal tube. Neonates were ventilated and their secretions were collected in a closed cuvette, which was immediately placed in ice and sent to the lab for analysis. The TA samples were centrifuged (400 rpm for 5 min).

### Analytical methods

#### Flow cytometry on TA

**Fig 1:**
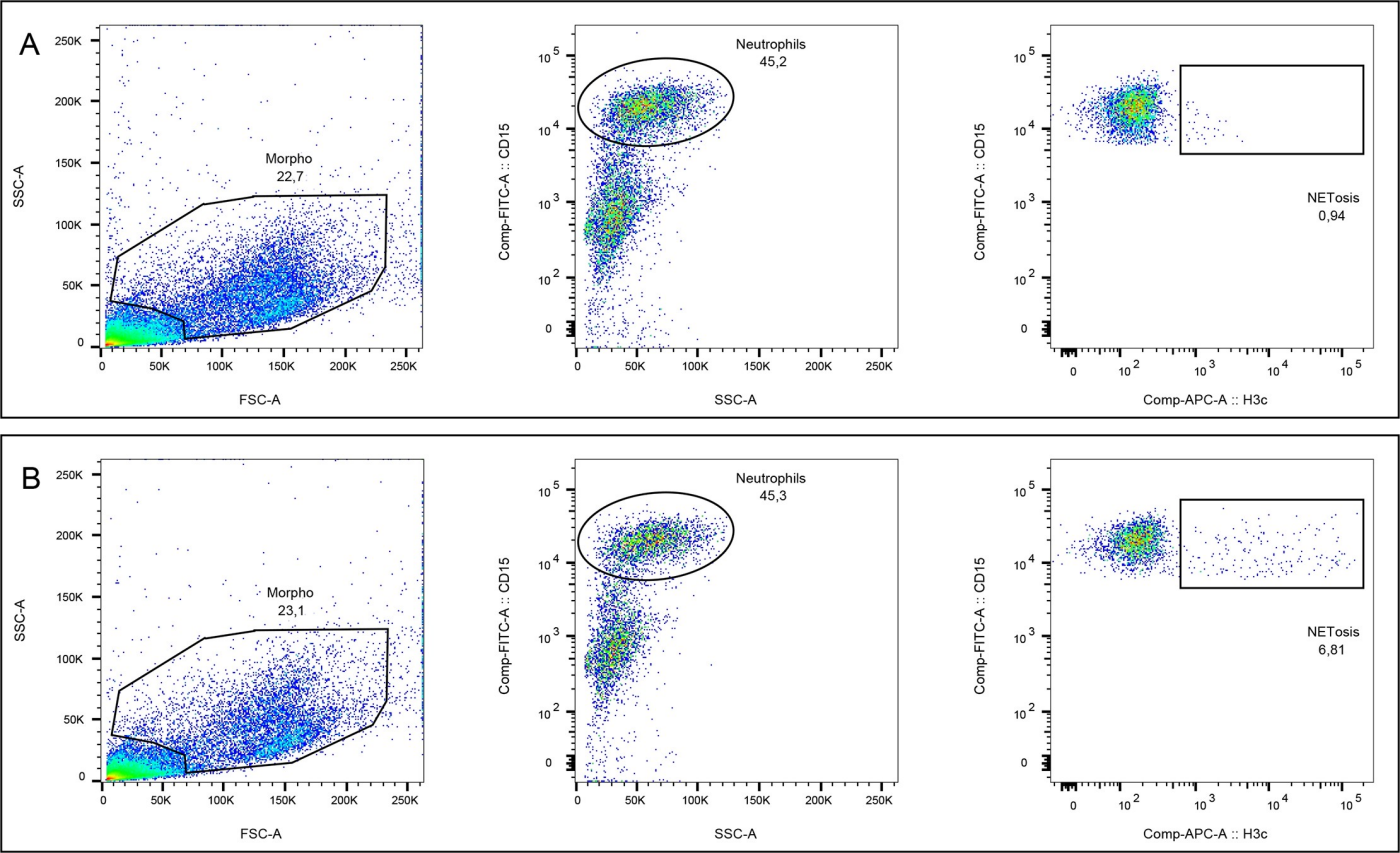
NETosis analysis in TAs from a newborn. Panel A shows the isotype control where mixed rabbit IgG were incubated, instead of anti-H3c, before adding AF-647 conjugated secondary antibody. Panel B shows the results for a representative newborn.

Seeded cells were marked and placed in the flow cytometer to assess overall cell lineages, the percentage of dead cells and neutrophils, and NETosis. Fig 1 shows the flow cytometric data regarding NETosis in the TA from a representative newborn. The supernatant was also stored and frozen to measure neutrophil elastase (NE), myeloperoxidase (MPO), and urea. Cells were suspended in PBS and distributed in cuvettes containing at least 20000 cells. Samples were marked with live stain (Live and Dead Aqua, Thermo Fisher Scientific), a mouse FITC anti-CD15 antibody diluted 1:500, and anti-histone H3 citrullinated antibody (Abcam-ab119553, Cambridge, UK), for 20 min at 4°C (an unmarked sample was also assessed for autofluorescence), followed by lavage with PBS at 2000 rpm for 2 minutes. Cells were then incubated with an AF657-conjugated anti-rabbit IgG antibody for 20 min at 4°C. For each antibody used, the fluorescence minus one or isotype antibody was used as a control. After lavage with PBS at 2000 rpm for 2 min, cells were newly seeded in 100 mL of PBS, read with the FACSCanto II flow cytometer (BD Bioscience), and analyzed with FlowJo software. To standardize the cells and biochemical components analyzed, urea was assessed in serum and TAs. Urea is assumed to spread independently of any changes in vascular permeability and active transporter mechanisms, so we corrected the results obtained by multiplying the level of cells or enzymes in TAs by the ratio of plasma urea to TA urea. During NETosis, there is a chromatin decondensation caused by histone hypercitrullination, followed by the release of nuclear DNA together with nuclear and granular bacteriostatic proteins, MPO and NE. In our analysis of NETosis in TAs, we analyzed the percentage of NETosis in the neutrophil population using the citrullinated H3 (H3c) specific antibody. H3c is exposed on the surface of neutrophils during NETosis, and it is considered a marker of this process. A CD15-specific antibody was used for neutrophil selection because testing for CD15 and CD16 in our experimental setting showed that eosinophils only express CD15, whereas neutrophils express both. Using these two markers, we found no eosinophils in TAs. We thus decided to use only CD15 as a marker for neutrophils in order to minimize the staining protocol, the number of fluorophores and, most importantly, the great sample viability.

#### ELISA analysis on TAs

Two commercial kits (ABcam, Cambridge, UK) were used to examine NE and MPO concentrations in TAs. A 96-well plate was covered with anti-NE or MPO antibodies. Controls and samples were added to the wells and incubated at room temperature. After lavage, a second avidin-conjugated antibody was added to the wells and incubated at room temperature for 1 h. The same procedure was used with streptavidin-HRP conjugate. Tetramethylbenzidine was added as the HRP substrate. A yellow stain changing from blue color was seen after adding the acid solution, and the yellow stain is proportional to the level of NE. (www.abcam.com). Similarly, urea was assayed using colorimetry with the KA 1652 kit from Abnova. (www.abnova.com).

#### Routine patient care

WBC counts were obtained using standard laboratory techniques with a Siemens ADVIA 2120 counter (when urgent) or a Sysmex XE-2100 (for scheduled tests), including absolute segmented neutrophil, eosinophil, and basophil counts, together with lymphocyte and monocyte differential counts. Antibiotics were administered prophylactically to all NICU newborn and stopped when sepsis-screening results were negative (based on total WBC counts, absolute neutrophil counts, C-reactive protein levels, and negative bacterial cultures). Newborn infants were diagnosed with early-onset sepsis if a culture yielded pathogenic bacteria or increased C-reactive protein levels. As a standard nasal continuous positive airway pressure (N-CPAP) system, we used the circuit made with Hudson prongs (Hudson Respiratory Care, Temecula, CA, USA) or a Vygon cannula (VYGON Italia srl—Gruppo Vygon, info@vygon.it) connected to a Babylog VN500 (Dräger Medical AG & Co. KGaA, Lübeck Germany) or heated and humidified high-flow nasal cannula (HHHNC) (http://www.neotechproducts.com/product-catalog/neotech-ram-cannula). N-CPAP pressure was adjusted routinely to 5–7 cmH_2_O, and the largest prongs that could fit easily into the nostrils were used in each infant. The N-CPAP treatment criteria were clinical signs of increased breathing effort (i.e. increased respiratory rate, retractions of the lower ribs and sternum, grunting) with a preserved respiratory function, and a F_iO2_ request no higher than 0.30, a P_aCO2_ no higher than 54 mm Hg (arterial blood) or 58 mm Hg (capillary blood), and a pH > 7.2. Most patients with an increased oxygenation demand may have needed a dose of surfactant. A Babylog VN500 was used for synchronized mechanical ventilation (SIMV) or high-frequency ventilation (HFV). Expiratory tidal volumes of 4–6 mL/kg of BW were allowed. End expiratory pressure began at 5–6 cmH_2_O, depending on the F_iO2_ and lung inflation. Inspiratory times of 0.30–0.40 s were allowed, with rates not to exceed 50 breaths/min. The NICU protocol establishes that arterial oxygen saturation, as measured by pulse oximetry, be maintained between 92% and 96%, with an arterial pH of at least 7.20, and moderate permissive hypercapnia with a partial pressure of carbon dioxide (P_aCO2_) of 40–55 mm Hg.

#### Statistical data analysis

An exploratory data analysis was performed using heatmaps [16]. In the heatmap, the dependence between pairs of variables was estimated with Spearman’s correlation coefficient in the case of continuous variables, while for dichotomous variables and continuous variables we used the Mann-Whitney test or t-test, depending on the data distribution, to establish whether different levels of the categorical variable were characterized by different medians or means of the continuous variable. Normal distributed data were assessed with the Shapiro-Wilk test (p-value >0.10). Since we were testing more than one hypothesis simultaneously, false discovery rate correction was applied to the heatmap, and significantly related pairs of variables were selected on the basis of the q-values [17].

Multivariate data analysis was applied to discover more complex relationships than those revealed by pairwise comparisons. Methods based on projection to latent structures (PLS) were used because the variables being measured were not independent, a strong correlation applied to the data. In PLS, the measured variables are linearly combined into so-called latent variables that can be investigated to ascertain the relationships existing between the data collected. Both regression and classification problems can be solved, depending on whether the outcome of interest is continuous or categorical. In the case of categorical outcomes, PLS becomes PLS-Discriminant Analysis (PLS-DA). More details on the use of PLS in data analysis can be found in Stocchero et al. [18]. Decision trees based on the RIPPER algorithm were also built to obtain simple decision rules. The predictive power of the multivariate models was estimated by means of 5-fold cross-validation (CV). The statistical data analysis was performed using R-packages and in-house R-functions implemented on the R 3.3.2 platform (R Foundation for Statistical Computing).

## Results

### Study A: Prospective assessment of WBC counts in mothers and newborn

#### Newborn enrolled

Thirty-nine newborn infants were involved in this prospective observational study. They were all intubated and on mechanical ventilation. Their neonatal data are summarized in Table 1 and the results of the data analysis based on Spearman’s correlation, t-test or Mann-Whitney test are given in Table 2.

**Table 1:** Study A: Details of the sample of newborn (n = 39) (median and range for continuous variables; n. (%) for Boolean variables).

Mother		Newborn	
Age, y	42 (20–42)	GA, wk^+d^	32^+ 4/7^ (23^+ 2/7^–41^+ 4/7^)
Medically-induced pregnancy	5 (12.8)	BW, g	1828.4 (545–3790)
Pre-eclampsia	4 (10.2)	M/F	21/18
PPROM	5 (12.8)	Antenatal steroids, 1 dose	6 (15.4)
Twins	8 (20.5)	Antenatal steroids >1 dose	21 (53.8)
Vaginal GBS positive	9 (23)	Fetal growth restriction	6 (15.4)
Antibiotics	7 (17.9)	Apgar score < 5 at 1 min	15 (38.4)
Cesarean section	27 (69.2)	NICU admission pH	7.21 (6.75–7.39)
Histological chorioamnionitis	11 (28.2)	Respiratory distress syndrome	28 (71.8)
		Early-onset sepsis	17 (43.6)
		PDA	23 (58.9)
		Surfactant: 1 to 3 doses	28 (71.8)
		Dead	4 (10.2)
		BPD	10 (25.6)

**Table 2:** Analysis of neonatal data: Spearman’s correlation coefficient ρ (p-value), t-test (p-value) or Mann-Whitney test (p-value).

Response	Variable	ρ	p-value	t-test or Mann-Whitney test	p-value	Difference
WBC at birth	BW	0.38	0.024			
	GA	0.37	0.024			
	antenatal steroids	-0.43	0.008			
	pre-eclampsia			0.017	0.018	pre-eclampsia < no pre-eclampsia
	mode of delivery			0.018	0.035	vaginal > cesarean section
WBC at sampling	BW	0.37	0.030			
Blood neutrophils at sampling	BW	0.40	0.024			
WBC days 1 to 3	pre-eclampsia			0.008	0.01	pre-eclampsia < no pre-eclampsia
Blood neutrophils days 1 to 3	BW	0.40	0.027			
TA neutrophils	BW	0.64	<0.001			
	GA	0.60	<0.001			

#### Association between prenatal and postnatal clinical features and mothers’ or newborn’s blood/TA WBCs

**Fig 2:**
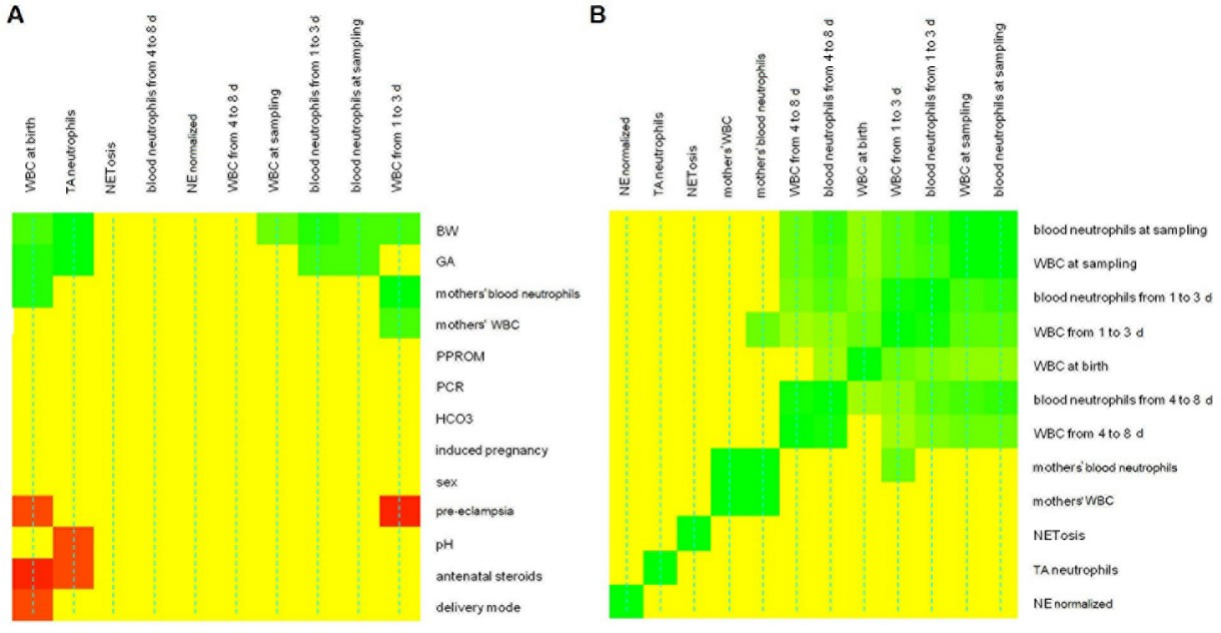
Heatmap obtained for blood and TA data, and pre- and postnatal data. Heatmap used to investigate the relationships between newborn’s blood data, maternal blood data, and newborn’s TA data. Green indicates a positive dependence, red a negative dependence, and yellow pairs of variables excluded on the basis of a false discovery rate.

The relationships between the newborn’s clinical features and their blood/TA data were investigated using heatmaps based on Spearman's correlation and the Mann-Whitney test. The threshold used for assessing significance was set to 0.15 on the basis of the false discovery rate. The heatmap is shown in Fig 2A (statistics with q-value >0.15 are colored in yellow, color code from red to green indicates negative and positive dependences). Higher WBC counts at birth coincided with higher newborn BWs and GAs, and higher mothers’ blood neutrophil levels, while they were lower the higher the dose of antenatal steroids administered. High WBC counts at birth were also observed in the absence of pre-eclampsia and for vaginal deliveries. The percentage of neutrophils in TAs was higher for higher BWs and GAs too, while it was lower for higher pH values and higher doses of antenatal steroids. Blood neutrophils at tracheal aspirates’ sampling, and at 1 to 3 days old correlated positively with BW and GA, while WBC counts at tracheal aspirates’ sampling, and at 1 to 3 days old were only higher for higher BWs. WBC counts at 1 to 3 days old were also higher if the mothers’ blood neutrophil concentrations and WBC counts were higher, and showed the highest levels in the absence of pre-eclampsia.

#### Association between mothers’ blood and newborn’s blood and TA WBC data

The relationships between the maternal and neonatal WBC and neutrophil levels, at the various time points, and NETosis were investigated using heatmaps based on Spearman’s correlations (Fig 2B). We only considered correlations with a q-value <0.10. Positive correlations are colored in green, those with a q-value >0.10 in yellow. No correlations emerged between the maternal and newborn blood and TA parameters, i.e. for NETosis, TA neutrophils or normalized NE (NEnorm). A moderate correlation (Fig 2A) was seen between the WBC and neutrophil levels of the mothers and their newborn in the first 3 days of life. The newborn’s WBC and neutrophil levels correlated moderately in the first 8 days of life (Fig 2B).

#### Predicting BPD and other clinical features from blood/TA neutrophils and NETosis

The variables considered were BW, GA, antenatal steroids, pH, pCO_2_, HCO_3_, base excess (BE), max C reactive protein levels from days 1 to 8, surfactant doses, severity of respiratory distress syndrome (RDS), delivery, premature pre-labor rupture of membranes (PPROM), pre-eclampsia, sex, medically-induced pregnancy, PDA, fetal growth restriction. No direct correlation was found between blood or TA neutrophils, or NETosis and BPD. A set of decision rules based on three variables—BW, antenatal steroids, and surfactant doses—was obtained using the RIPPER algorithm. The classification error calculated from the 5-fold coefficient of variation (CV) was 14%. Excluding antenatal steroids and surfactant doses, the rule “a BW of less than 1100 g is associated with the onset of BPD” was obtained, with a classification error in 5-fold CV of 20%. It is worth noting that GA and BW correlated strongly (r2 = 0.85, p-value<0.001), and GA could substitute BW in the decision rule, obtaining similar results (the rule “a GA of less than 203 days is associated with the onset of BPD” produced a classification error in 5-fold CV of 20%).

#### Study B: Retrospective examination of monocytes and macrophages in previously-enrolled newborn

The study groups and the methods for measuring monocytes and macrophages were as reported in Milan et al. [15]. Briefly, we enrolled a total of 108 newborn infants with a mean GA of 29 wk ^+1/7d^ (SD = 3 wk ^+1/7 d^), a mean BW of 1274.1 g (SD = 648.6 g), antenatal steroids in 71.3% of cases, and RDS in 97.2%, HCA in 22.3%, and BPD in 22%.

#### Results from lines of monocytes and macrophages for GA-matched newborn

**Fig 3:**
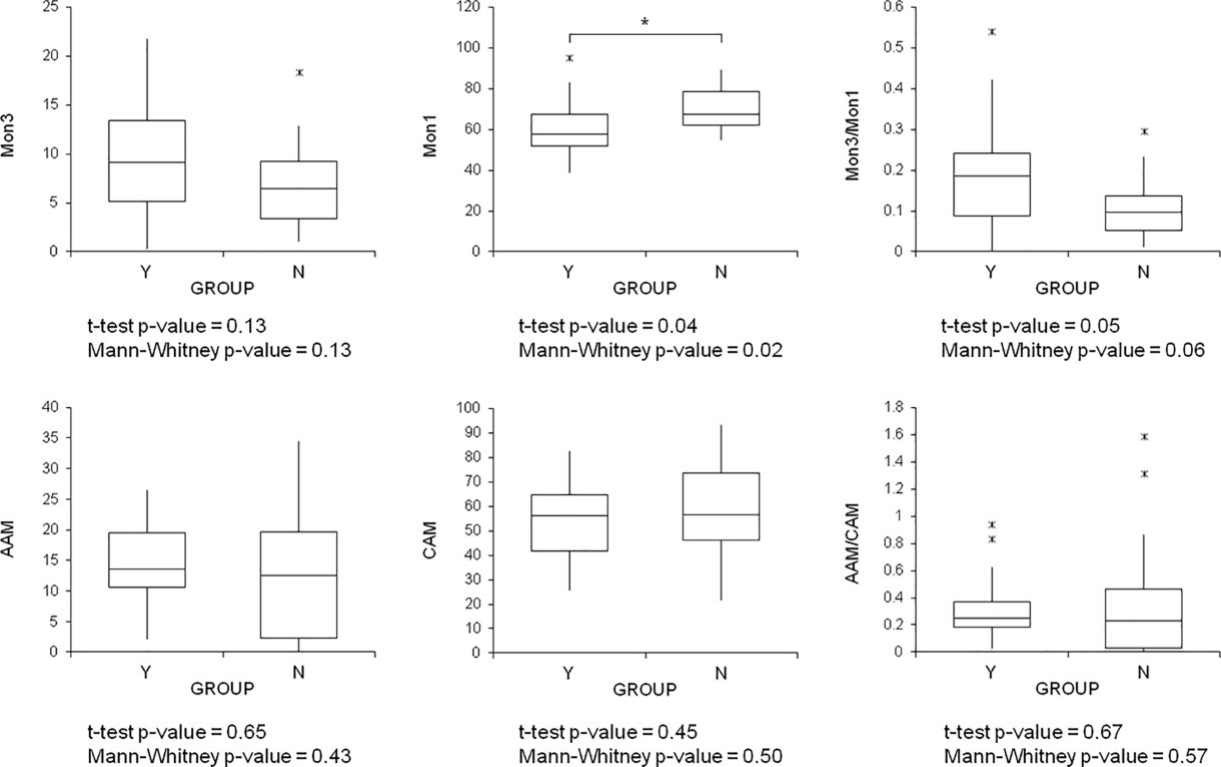
Box plots of predictors and related p-values for the t-test and Mann-Whitney test; Y indicates the group that developed BPD, N the group that did not. Asterisks (*) and horizontal bars indicate intergroup differences; p<0.05 was considered the limit for statistical significance. Box indicates medians with 25^th^ and 75^th^ percentiles, and whiskers indicate 5^th^ and 95^th^ percentiles; >95^th^ or <5^th^ percentiles are also shown (x).

A subset of 32 newborn infants extracted from the whole dataset included 16 who developed BPD and 16 matched for GA (from 25 to 32 weeks) who did not. A PLS-DA model (autoscaling, 1 latent variable) was able to distinguish between these two groups, with a 28% classification error in 5-fold CV, and a receiver operating characteristic (ROC) curve showing an area under the curve (AUC) of 0.68 in 5-fold CV. Box plots of the predictors are shown in Fig 3. Lower levels of Mon1 and a higher Mon3/Mon1 ratio were seen in the newborn who would develop BPD. There were no differences among the macrophage phenotypes in the TAs, and the latent variable was not found correlated with GA.

**Fig 4:**
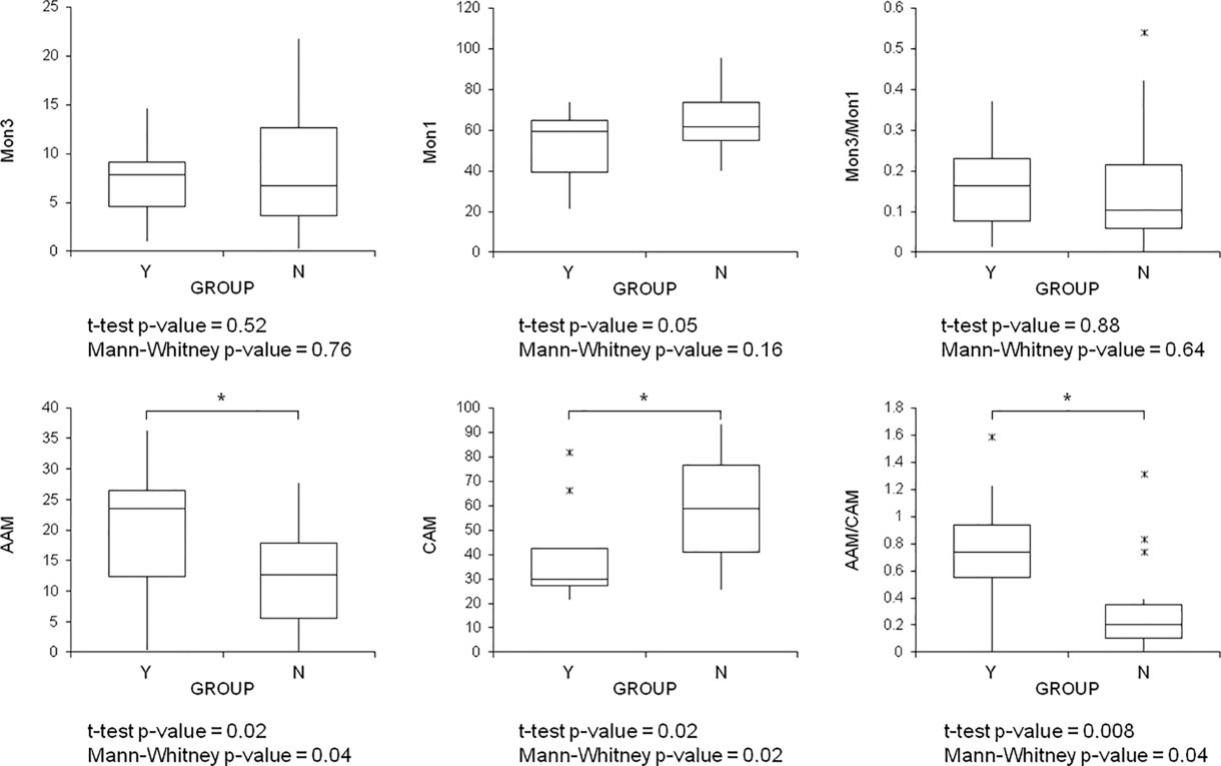
Box plots of predictors and related p-values for the t-test and Mann-Whitney test; Y indicates the group with maternal HCA, N the group without it. Asterisks (*) and horizontal bars indicate intergroup differences; p<0.05 was considered the limit for statistical significance. Box indicates medians with 25^th^ and 75^th^ percentiles, and whiskers indicate 5^th^ and 95^th^ percentiles; >95^th^ or <5^th^ percentiles are also shown (x).

Another subset extracted from the whole dataset consisted of 29 newborn forming two groups matched for GA (from 23 to 32 weeks), 9 with and 20 without maternal HCA. A PLS-DA model (autoscaling, 1 latent variable) was able to distinguish between the two groups with a classification error of 21% in 5-fold CV, and a ROC curve with an AUC of 0.78 in 5-fold CV. The latent variable did not correlate with GA. Box plots of the predictors are shown in Fig 4. HCA increased the TA-AAM and the AAM/CAM ratio, while it reduced the TA-CAM, and also the Mon1 in the blood. Finally, a subset of 40 newborn extracted from the whole dataset consisted of two groups matched for GA (from 25 to 35 weeks), 26 with and 14 without PDA. No reliable discriminant models were obtained to demonstrate any relationships between PDA and macrophage or monocyte phenotypes.

## Discussion

Our studies aimed to identify prematurity-related changes in cellular innate immunity in blood and TAs, and to identify any association with the onset of BPD. The results of Study A confirmed previous findings regarding the immaturity of the preterm-born infant’s immune system, with a reduced pool of monocytes and more neutrophils. The latter differences are greater for higher GAs and BWs of the newborn [19, 20]. Neutrophils in TAs were also found to correlate with BW and GA, like the possible immunological maturation happens also for lung inflammatory cells. The association between mothers’ and newborn’s blood neutrophil levels is known and can be explained by a transplacental passage of inflammatory stimuli [21]. Our analysis failed to reveal any specific correlation between maternal HCA and newborn WBC, probably because other pathological conditions such as PPROM or apparently spontaneous premature labor could act as stimulators of both maternal and fetal myelopoiesis.

On the other hand, lower WBC levels were found associated with pre-eclampsia, which is a known cause of leukopenia and neutropenia [22, 23]. It has been suggested that leuko-neutropenia could be the result of a lower production and a weaker response to growth factors in the offspring of hypertensive mothers. Another possible explanation is that uteroplacental failure and consequent chronic hypoxia could lead to an excessive erythropoiesis, thus hampering granulopoiesis [24].

As previously reported, higher WBC counts were identified in newborn infants after vaginal delivery [25]. Interestingly, functional neutrophil deficits (chemotaxis and chemokinesis) have also been demonstrated in preterm babies born by cesarean section, possibly as a consequence of anesthetic drugs administered to the mother [26]. In our cohort of newborn infants, antenatal steroid administration was associated with lower blood WBC counts in the newborn. Although steroids have been shown to have suppressant effects on the offspring’s immune system [27], our finding is in contrast with previous reports of higher leukocyte and neutrophil counts in premature infants exposed to antenatal betamethasone [28].

The lack of any correlation between NETs and neutrophils in TAs probably stems from the balance between suicidal [29] and vital [30] NET pathways. The release of NETs can be seen in a phase involving more neutrophil death than survival, which would lend an apoptotic and necrotic “meaning” to NET levels. This would explain why we found NET and neutrophil levels in TAs unrelated.

Our search for an early WBC imbalance as a marker of the development of BPD was unsuccessful: no direct correlations emerged between blood or TA neutrophil counts, or NETosis and BPD.

We could nonetheless identify some known risk factors of BPD development in our population: a BW of less than 1100 gr, or a GA of less than 203 days correlated strongly with the onset of BPD. This underscores how preventing preterm delivery should be the main objective for the prevention of BPD.

On the other hand, the retrospective part of this study (study B) revealed some promising details regarding lower Mon1 levels and a higher Mon3/Mon1 ratio as possible factors involved in BPD development, or as markers of its onset. Although extremely preliminary, these findings suggest that an early polarization of monocytes towards a classical activation may help to protect against BPD. A higher Mon3/Mon1 ratio in the blood presumably led to an altered endothelial function involving the migration of monocytes into the intima [31], where they differentiate into macrophages. This pathway may also be responsible for the disrupted angiogenesis seen in BPD in experimental models [32], and humans [33–35]. http://dx.doi.org/10.1016/S2352-4642(18)30181-0 The reduction of Mon1 noted in HCA patients also points to a pathogenic hypothesis for the relationship between HCA and BPD. These data warrant further investigation.

Lastly, PDA appears to be involved in BPD development without modifying inflammatory cell levels, and duct patency does not seem to be predictable from blood and TA monocyte-macrophage profiles studied here.

A limitation of our work lies in the revalidation of our metadata because the whole group of patients had already been partially studied (with no results contrasting with those reported here). The relationship found here in the prospectively-studied sample mainly concerns the “effect” of GA on WBC and neutrophil levels in the blood or TAs. This could also justify differences relating to delivery mode (vaginal vs cesarean section) and pre-eclampsia compared with normal pregnancy.

In conclusion, studying the innate and adaptive immune system has potential as a forward-looking approach to BPD [34], especially with a view to developing innovative strategies to prevent or treat the disease, based on stem cells or their secretome [36–38] instead of surfactants and steroids, or the microbiome [36, 39]. Surprisingly, lower levels of neutrophils were seen in the TAs from the newborn infants with the lowest GAs and BWs. No correlations between neutrophils and NETs in TAs emerged with regard to the other variables. Since the literature supports a role for the inflammasome and inflammatory cells in the lung, as well as for proinflammatory cytokines and soluble adhesion molecules [1], our findings regarding NETs and neutrophil levels in TAs (i.e. our failure to identify any predictors of BPD) was unexpected, and may largely reflect the evolving pattern of the immune cells considered. The paths of inflammation and immune modulation are not yet fully understood, and investigations on perinatal lung development in clinical settings are worth pursuing.

## Supporting information

S1 Data Laboratory data of the study A patients. (XLSX)
